# Estimation of Sarcopenia Indices in Women from Saudi Arabia in Relation to Menopause and Obesity: Cross-Sectional Comparative Study

**DOI:** 10.3390/jcm12206642

**Published:** 2023-10-20

**Authors:** Nouf Aljawini, Syed Shahid Habib

**Affiliations:** 1 Department of Physiology, College of Medicine, King Saud University, Riyadh 11416, Saudi Arabia; naljawini@ksu.edu.sa (N.A.);; 2 Department of Community Health Sciences, College of Applied Medical Sciences, King Saud University, Riyadh 11433, Saudi Arabia; naljawini@ksu.edu.sa

**Keywords:** sarcopenia, probable sarcopenia, EWGSOP2, handgrip strength, cut-off, women, menopause, obesity, Saudi Arabia, prevalence

## Abstract

Sarcopenia prevalence depends on the definition, and ethnicity must be considered when setting reference values. However, there is no specific cut-off for sarcopenia in Saudi women. Accordingly, we aimed to establish a cut-off value for sarcopenia in Saudi women. We determined the prevalence of sarcopenia in terms of low handgrip strength (HGS) in postmenopausal women using the EWGSOP2 value, redefined a specific cut-off for low HGS derived from Saudi premenopausal women, re-determined the prevalence of low HGS using the new cut-off, and analyzed the proportion of low HGS in women with obesity compared to those without obesity. Following EWGSOP2 guidelines, we defined probable sarcopenia and set new HGS values. We assessed HGS and body composition in 134 pre/postmenopausal women. Probable sarcopenia prevalence was calculated using EWGSOP2’s HGS of 16 kg and new cut-offs from young premenopausal women without obesity. HGS 10 and 8 kg cut-offs were calculated from premenopausal Saudi women’s mean −2 SDs and mean −2.5 SDs. Using the HGS 16 kg cut-off, sarcopenia prevalence was 44% in postmenopausal and 33.89% in premenopausal women. Applying the new HGS 10 kg and 8 kg cut-offs, the prevalence was 9.33% and 4%, respectively, in postmenopausal and 5% and 3.40%, respectively, in premenopausal women. Women with obesity had a higher proportion of low HGS across all cut-offs. We suggest that EWGSOP2 cut-offs may not be adaptable for Saudi women. Considering body composition differences between Saudis and Caucasians, our proposed HGS cut-offs appear more relevant.

## 1. Introduction

Sarcopenia is a progressive skeletal muscle syndrome that accelerates muscle mass and function loss, increasing mortality risk, falls, fractures, and physical disability [1]. Rosenberg was the first to come up with the term “sarcopenia” in 1989 to refer to the loss of muscle mass after comparing the lean body mass of an older woman’s thigh to that of a younger woman. It is derived from the Greek words “sarx” (flesh) and “penia” (loss) [2]. Over the last 30 years, the research has evolved from evaluating muscle mass to combining muscle strength and physical function into a more comprehensive definition, described as losing muscle and strength [1]. In 2016, sarcopenia was officially identified as a muscle disease in the International Classification of Diseases (ICD-10: M62 (84)) [3].

Handgrip strength (HGS) is a valuable muscle strength index recommended to diagnose sarcopenia [1]. As muscle strength predicts accidents, fractures, and all-cause mortality, low muscle strength is considered a more significant indicator of sarcopenia than low muscle mass [4,5]. The European Working Group on Sarcopenia in Older People 2 [EWGOSP2] recently proposed a new definition of sarcopenia, focusing on handgrip strength (HGS) rather than muscle mass as a primary criterion of sarcopenia [1]. EWGSOP2 defined cut-off points for sarcopenia indices to harmonize investigations. They suggested using HGS cut-offs lower than 27 kg and 16 kg for males and females, respectively, derived from large-scale studies of the British population [1,6].

Sarcopenia-related cut-off values might differ by the demographics, especially among height and muscle strength-based metrics. EWGSOP2 pointed out that it may be better to employ population-specific cut-offs if available. As the prevalence of low HGS changes along with the HGS cut-off, for instance, the HGS cut-offs suggested in EWGSOP1 (30/20 kg) are significantly higher than the EWGSOP2-recommended HGS cut-offs (27/16 kg) for detecting probable sarcopenia. Accordingly, EWGOSP2 suggested using HGS cut-offs lower than 2.5 standard deviations from the mean value of the HGS of the young adult in a specific population [1].

Aging, sociodemographic characteristics, lifestyle, and multiple medical conditions contribute to the development of sarcopenia. Previous studies on the etiology of sarcopenia have produced inconsistent and disputed findings [7]. Age significantly determines women’s physical function; however, menopause may have additional effects beyond age alone and is associated with decreased estrogen. Women who reach menopause at an earlier age have greater exposure to the adverse consequences of a decline in estrogen levels, which increases cardiovascular risk and osteoporosis due to decreased physical function [8,9]. Estrogen levels decline in women after menopause at around 50 years, decrease intensely within six months around menopause, and continue for three years after menopause [10]. Multi-site studies in postmenopausal women have shown that those with premature menopause (under 40 years) have significantly weaker grip strength compared to women who underwent menopause at a later age [11,12,13].

It is likely that the cut-off of low muscle strength used in premenopausal women is not transposable to postmenopausal women. Although data are limited, it appears that women post menopause present a specific change in body composition with menopause. We recently reported that menopause in Saudi women was associated with changes in body composition; postmenopausal women have lower muscle mass and higher BMI and body fat percentage than premenopausal women [14]. This observation raised two questions: Shall we apply the same cut-off for premenopausal and postmenopausal women to detect low muscle strength? Are the commonly used HGS cut-offs applicable to Saudi women?

Cut-off values for sarcopenia have not been defined in Saudi women, which is essential since that body composition varies significantly among ethnic groups [15]. The disparity in sarcopenia prevalence based on different cut-points emphasizes the importance of applying ethnic-specific cut-offs in clinical practice [16]. Using the same HGS cut-off in premenopausal and postmenopausal women may result in sarcopenia being underdiagnosed or over diagnosed. This report aimed to estimate the HGS cut-offs in postmenopausal women derived from premenopausal Saudi women as a reference population following the EWGSOP2′s recommendation and compare it to the EWGSOP2 values. 

We hypothesized that menopausal transition is associated with significantly lower HGS. This comparative study was designed to determine the prevalence of low HGS, a criterion for sarcopenia diagnosis, in Saudi premenopausal and postmenopausal women using the EWGOSP2 cut-offs, develop a new algorithm redefining low HGS in Saudi postmenopausal women with a new HGS cut-off based on data from premenopausal Saudi women, re-determine the prevalence of low HGS by applying this new cut-off in postmenopausal women in this population, and analyze the proportion of low HGS in women with obesity compared to women with non-obesity.

## 2. Material and Methods

### 2.1. Study Design and Settings

This cross-sectional study compared sarcopenia and body composition indices between premenopausal and postmenopausal women, and it was reported following the Strengthening the Reporting of Observational Studies in Epidemiology (STROBE) recommendations [17]. The research was conducted in the Physiology Department at King Saud University (KSU) and King Saud University Medical City (KSUMC) in Riyadh from March 2022 to August 2023. Ethical approval was obtained from The Institutional Review Board of KSU College of Medicine (No. E-21-5998), and all women provided written informed consent. The study followed the Helsinki Declaration guidelines for experiments involving human beings [18].

### 2.2. Participants and Protocols

Women who visited the KSUMC outpatient clinics and completed the study procedures were eligible to participate. One hundred thirty-four women aged 18 to 83 were recruited and grouped into premenopausal and postmenopausal groups based on their menopausal status. Medical history and menopausal status were confirmed through medical records and interviews with questionnaires. All women provided menstrual history information through semi-structured interviews. Menopausal status was determined based on their bleeding pattern and categorized as a binary variable, with premenopausal and postmenopausal groups defined using the Stages of Reproductive Aging Workshop criteria [19]. Women were classified as premenopausal if they had regular menstrual cycles and postmenopausal if they had not had a menstrual cycle for over 12 consecutive months. Pregnant women, those with acute medical illnesses or acute treatment that affects body composition, or those with physical handicaps that could interfere with bioelectrical impedance analysis and strength measurements were excluded. Participants with a 50 kg/m^2^ BMI or higher were also excluded. Obesity was defined as a BMI of 30 kg/m^2^ or higher [20]. Thirty premenopausal women with non-obesity constituted the “healthy young adult reference group” control group to calculate handgrip strength cut-off points to identify low grip strength in postmenopausal women, known as the ‘older adults’ group. 

### 2.3. Sarcopenia Definition

The sarcopenia index is described as probable sarcopenia, as defined by the EGWSOP2 consensus. Low muscle strength, detected by low handgrip strength (HGS), is the primary outcome for determining probable sarcopenia. The algorithm of the EWGSOP2 was followed to diagnose probable sarcopenia, and the EWGSOP2 female cut-off of <16 kg was applied [1].

### 2.4. Currently Used Cut-Offs for the Definition of Low HGS

We used a digital dynamometer to measure handgrip strength, and we used an EGWSOP2 cut-off of HGS < 16 kg [1] to characterize the cohort into probable sarcopenia and non-sarcopenia groups.

### 2.5. Validation of Low HGS from Saudi Premenopausal Reference Population

Applying the EGWSOP2 consensus recommendation [1], we utilized the HGS data of premenopausal Saudi women with a BMI less than 30 kg/m^2^ as the ‘‘reference group’’ to redefine the cut-off for low HGS in postmenopausal Saudi women. The cut-off points for low grip strength were derived from the mean minus 2 standard deviations and the mean minus 2.5 standard deviations of the premenopausal reference group, and the figures were rounded for practicality.

### 2.6. Study Procedures

#### 2.6.1. Handgrip Strength (HGS) Measurements

To evaluate muscle strength, handgrip strength (HGS) was measured using a digital hand dynamometer (Digital grip strength dynamometer, T.K.K 5401, Takei Scientific Instruments Co., Ltd., Niigata, Japan). The testing was conducted with the participants sitting, their arms alongside the trunk, and their elbows flexed at 90°. Three maximum trials were performed with the dominant hand, with a 1 min interval between testing. The highest value was used in the analysis.

#### 2.6.2. Appendicular Skeletal Muscle Mass Measurements

The surrogate sarcopenia index for muscle mass, defined as appendicular skeletal muscle mass (ASM) [1], was estimated by summing the ASM of the arms and legs, as described by Heymsfield et al. [21]. The ASM index (ASMI) was then calculated as ASM per height squared [ASMI; kg/m^2^]. We used bioelectrical impedance analysis to estimate the lean mass of legs and arms.

#### 2.6.3. Anthropometrics Measurement and Body Composition Evaluation

We measured anthropometrics and body composition following the study protocol’s standardized operating procedures.

The participants’ weights and heights were measured using a Seca electronic scale and stadiometer (Seca 576; SECA CORP, Hamburg, Germany). They were weighed standing without shoes and in light clothing, with an approximation of 0.1 kg, and their height was recorded to the nearest 0.1 cm. The body mass index (BMI) was calculated by dividing the weight in kilograms by the square of the height in meters (kg/m^2^) and categorized using the World Health Organization standards (WHO) [20]. Obesity is a BMI of 30 kg/m^2^ or higher [20]. Body fat percentage (%BF) was chosen as a surrogate marker for total body adiposity. The participants’ body composition was estimated using a Tanita bioelectrical impedance analysis (BIA) device (Tanita MC-980, Tokyo, Japan). This analyzer measured the electrical impedance of each of the body’s five segments (left arm, right arm, trunk, left leg, and right leg) at six frequencies (1 kHz, 5 kHz, 50 kHz, 250 kHz, 500 kHz, and 1 MHz) to determine the proportion of tissues, such as protein, fat, muscle, mineral, and body water content. The analyzer used an 8-electrode system, in which current was supplied from the toes of both feet and the fingertips of both hands while the voltage was measured on the heel of both feet and hands. The measurements were performed barefoot on a footplate with separate electrodes and by holding the right- and lefthand electrodes. The segmental lean analysis was conducted in five different body sectors (arms, trunk, and legs) to evaluate body composition further. The body fat percentage (%BF) and fat-free mass (FFM) were estimated using BIA, as they offer convenience and performance that have been validated in previous studies compared to dual X-ray absorptiometry [22]. The measurements were highly reproducible, with an intra-device coefficient of variation ranging from 0.8 to 0.9% and an inter-device coefficient of variation of 0.1% [23]. A trained researcher conducted all tests following our Standard Operating Procedures (SOPs).

### 2.7. Statistical Analysis

We calculated the mean differences of relevant parameters and standard deviations from similar studies using the online software OpenEpi 3.0 (https://www.openepi.com/SampleSize/SSMean.htm, accessed on 1 March 2022) to estimate the sample size [24]. It was observed that a sample size of 134 would be appropriate with 80% predictive power and a 95% confidence interval. Additionally, we searched in the literature and found some studies with similar designs and sample sizes [25]. We used Statistical Package for Social Sciences (SPSS) version 20.0 (IBM Corp., Armonk, NY, USA) for data entry and analysis. Subject characteristics were described as proportions for categorical variables and mean ± Standard Deviation (SD) for quantitative variables. Kolmogorov–Smirnova and Shapiro–Wilk tests were used to determine whether the data followed a normal distribution. Non-parametric tests, such as the Mann–Whitney test for two groups and the Kruskal–Wallis test for more than two groups, were used for skewed data. Group comparisons were made using Student’s *t*-tests, and for multiple group comparisons, analysis of variance (ANOVA) was used for normally distributed data. We performed post hoc analysis using Bonferroni multiple group comparisons, and the significant *p* values were recorded between different groups. Categorical variables are represented as numbers with percentages and analyzed using the chi-square test. All *p*-values were two-tailed. A *p*-value less than 0.05 was considered statistically significant.

## 3. Results

### 3.1. Sample Characteristics

**Figure 1:**
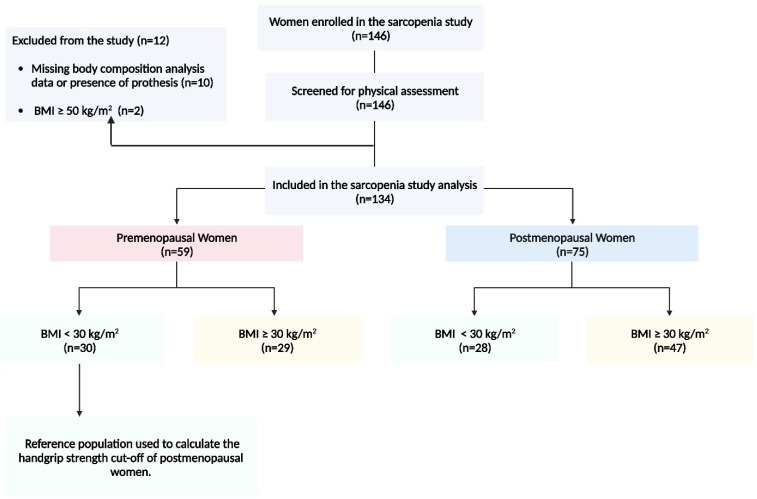
Participant flow diagram. Note: Participant flow diagram illustrating the number of women enrolled in the sarcopenia study assessed for eligibility, screened for physical assessment, and included in the sarcopenia study analysis according to menopausal status and obesity categories. Abbreviations: BMI: body mass index.

The sample included 134 women with an age distribution from 18 to 82 years. Women were divided into two groups according to their menopausal status. Figure 1 illustrates the study flowchart. Premenopausal women constituted the “the young women group” (*n* = 59; mean age 42.63 ± 8.25 years), while postmenopausal women (*n* = 75; mean age 60.95 ± 6.40) constituted the “the older adults group”. Premenopausal and postmenopausal groups were subdivided into two subgroups according to the absence or presence of obesity, with a body mass index (BMI) of 30 kg/m^2^ used as the cut-off point for determining obesity. Thirty premenopausal women with non-obesity constituted the “healthy young adult reference group” control group to calculate the handgrip strength cut-off points to identify the low grip strength of the postmenopausal women “older adults’ group”. Regarding the primary variable evaluated, the handgrip strength’s mean (standard deviation) was 17.15 (4.79) kg.

### 3.2. Clinical Parameters Associated with Menopausal Status and Obesity

The baseline anthropometric and body composition parameters and the comparison between pre- and postmenopausal groups are summarized in Table 1. Adiposity indices were significantly higher in postmenopausal women than in premenopausal women, with no statistical differences between the groups in fat-free mass (FFM). Statistically significant differences in body composition indices and the comparison between groups according to obesity and menopause are summarized in Table 2. Premenopausal women with non-obesity, who form the control group “healthy young adult reference group”, showed systematically lower BMI, %BF, FM, and FFM than postmenopausal women “older adults”, regardless of obesity status.

### 3.3. Sarcopenia Indices Associated with Menopausal Status and Obesity

The study examined handgrip strength (HGS) as the primary outcome to evaluate sarcopenia in Saudi women according to menopausal status and obesity. As shown in Table 1, the average HGS in the total sample was 17.15 (SD ± 4.79) kg, and postmenopausal women have significantly lower HGS than the premenopausal group (postmenopausal 16.39 ± 4.79; premenopausal 18.12 ± 4.65 kg; *p* = 0.037). Furthermore, the study looked at the differences in HGS between the menopausal group regarding the absence or presence of obesity, as illustrated in Table 2. HGS was the highest in the premenopausal women with non-obesity “control group”, with a mean of 18.83 (SD ± 4.45) kg; however, the difference between the groups was not statically significant.

In terms of appendicular skeletal muscle (ASM) and appendicular skeletal muscle index (ASMI), the mean value of the total sample was 18.66 (SD ± 2.77) kg for ASM and 6.80 (SD ± 0.76) kg/ht^2^ for ASMI, with was no statistical differences between premenopausal and postmenopausal women, as shown in Table 1. When categorizing the cohort into four groups based on obesity and menopausal status, the analysis showed statistical differences in ASM and ASMI, as presented in Table 2. Premenopausal and postmenopausal women with non-obesity have lower mean values of ASM and ASMI levels than those with obesity.

### 3.4. Prevalence of Probable Sarcopenia (Low HGS) in Saudi Women Using EWGSOP2 Cut-Off

Applying the EWGSOP2 cut-off, we calculated the prevalence of probable sarcopenia in Saudi women. The EWGSOP2 consensus specifies that HGS < 16 kg in women characterizes probable sarcopenia, while HGS > 16 kg indicates non-sarcopenia [1]. Probable sarcopenia was present in 39.55% of our total sample, as shown in Table 3. We analyzed the proportion of premenopausal and postmenopausal women among sarcopenia subjects. The postmenopausal group comprised 62.26% of the sarcopenia group, while the premenopausal group comprised only 37.73%, as illustrated in Table 3.

### 3.5. Definition of an Adapted Cut-Off for Detecting Low Handgrip Strength in Saudi Postmenopausal Women

We used the Saudi premenopausal women with non-obesity data “reference group” to redefine the Saudi postmenopausal newly adapted cut-off of low HGS, as shown in Table 4.

### 3.6. Comparing the Prevalence of Probable Sarcopenia Using EWGSOP2 Cut-Offs versus the Newly Defined Cut-Off According to Menopausal Status in Saudi Women

**Figure 2:**
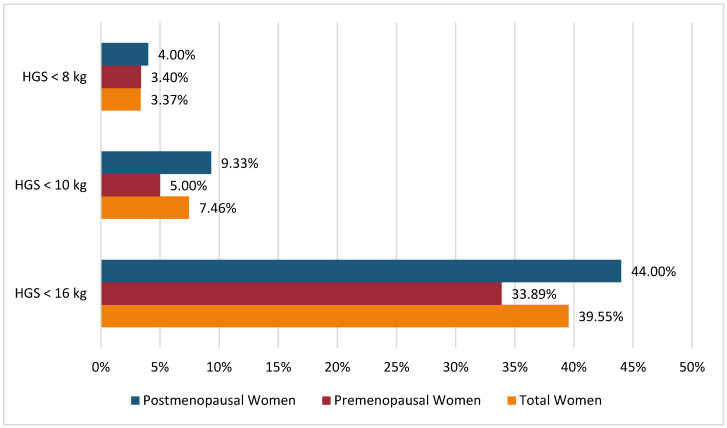
Comparison of the prevalence of probable sarcopenia according to menopausal status using EWGSOP2 cut-off and newly determined cut-offs. Note: Data are presented as percentages (%). HGS < 16 kg represents the EWGSOP2 cut-off to identify probable sarcopenia in women [1]; HGS < 10 kg represents newly derived cut-offs calculated from the mean minus 2 SD of the premenopausal Saudi women reference population; HGS < 8 kg represents newly derived cut-offs calculated from the mean minus 2.5 SD of the premenopausal Saudi women reference population. Abbreviations: HGS: handgrip strength; EWGSOP2: European Working Group on Sarcopenia in Older People 2. Chi-square *p*-value is >0.05.

The prevalence of low handgrip strength (HGS), which is indicative of probable sarcopenia, among the same Saudi postmenopausal women was found to be 9.33% (*n* = 7) and 4% (*n* = 3) when using HGS cut-offs of 10 kg and less than 8 kg, respectively, based on a reference group of Saudi premenopausal women. However, when an HGS cut-off of 16 kg was employed, the prevalence of low HGS increased to 44% (*n* = 33). In Figure 2, we illustrated the disparities in the prevalence rates of low HGS among the total sample, premenopausal and postmenopausal women, by employing the EWGSOP2 cut-off versus the newly defined cut-offs derived from the Saudi population. However, employing each HGS cut-off, the differences in menopausal group percentages were not statistically significant.

### 3.7. Prevalence of Low HGS (Probable Sarcopenia) in Saudi Women with Obesity Compared to Those without Obesity Using Different HGS Cut-Offs

**Figure 3:**
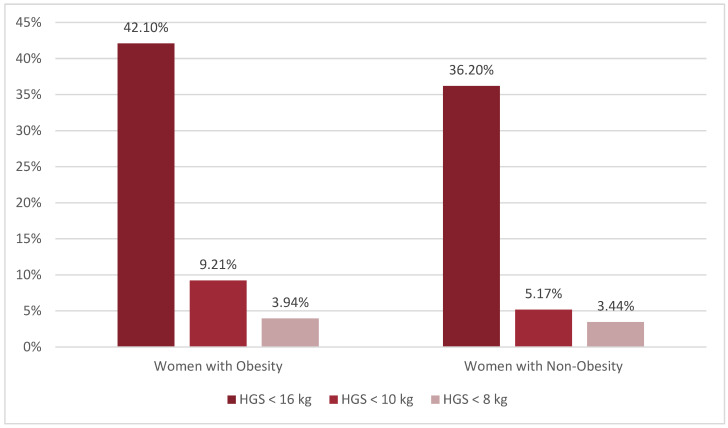
Comparison of the prevalence of probable sarcopenia according to obesity status using currently used and newly determined cut-offs. Note: Data are presented as percentages (%). HGS < 16 kg represents the EWGSOP2 cut-off to identify probable sarcopenia in women [1]; HGS < 10 kg represents newly derived cut-offs calculated from the mean minus 2 SD of the premenopausal Saudi women reference population; HGS < 8 kg represents newly derived cut-offs calculated from the mean minus 2.5 SD of the premenopausal Saudi women reference population. Abbreviations: HGS: handgrip strength; EWGSOP2: European Working Group on Sarcopenia in Older People 2. Chi-square *p*-value is >0.05.

When dividing our sample into two groups based on the absence or presence of obesity using a BMI cut-off of 30 kg/m^2^, as shown in Figure 3, we analyzed the distribution of probable sarcopenia between the groups using different HGS cut-offs. The prevalence of low HGS is higher in women with obesity than among women without obesity. When the EGWSOP2 HGS cut-off of 16 kg was applied to our sample, 42.10% (*n* = 32) of women with obesity and 36.20% (*n* = 21) of women without obesity had probable sarcopenia. However, when the newly specified HGS cut-off of 10 kg was used, the prevalence of likely sarcopenia reduced to 9.21% (*n* = 7) in women with obesity and 5.17% (*n* = 3) in women without obesity, as shown in Figure 3. Using a cut-off of 8 kg, the probable sarcopenic prevalence was comparable between women with obesity and without obesity, with rates of 3.94% (*n* = 3) and 3.44% (*n* = 2), respectively. However, the differences between the percentages of the obesity and non-obesity groups were not statistically significant using each HGS cut-off.

## 4. Discussion

The lack of uniform diagnostic criteria hinders sarcopenia prevalence and health impact estimates. Based on our research, this report is the first to provide Saudi-specific cut-offs for low handgrip strength, a primary criterion for diagnosing sarcopenia. These cut-offs were derived from young Saudi reference women considering menopausal status. We also evaluated the prevalence of sarcopenia when internationally reported cut-offs were used versus the newly calculated cut-offs. Based on the diagnostic criteria employed, we observed a considerable discrepancy in the prevalence of sarcopenia. Specifically, our proposed cut-off values of low handgrip strength (HGS), 10 kg and 8 kg, which we derived from Saudi premenopausal women, classified 9.33% and 4% of Saudi postmenopausal women, respectively, as sarcopenic. These values were lower than the internationally published cut-offs, which resulted in 44% of postmenopausal Saudi women being classified as sarcopenic when the internationally published cut-off of 16 kg was used.

Probable sarcopenia is low muscle strength without non-muscular conditions that affect muscle strength, such as stroke, peripheral conditions, balance disorders, or depression [1]. According to the revised European Working Group on Sarcopenia in Older People, an HGS of <27 kg for men and <16 kg for women is considered low and is derived from a comprehensive British study [1]. Dodds et al. analyzed data from 12 general population studies involving nearly 50,000 participants aged 4–90 years and defined low grip strength as 2.5 standard deviations below the highest mean HGS value for each sex. The maximum mean values for grip strength were reported as 51.9 (SD ± 9.9) kg for males and 31.4 (SD ± 6.1) kg for females, with both peaks occurring at 32 years of age [6]. The round values for low HGS were 27 and 16 kg in males and females, respectively, and the grip strength cut-offs that were two standard deviations below the peak mean of HGS for each group were 32 kg for males and 19 kg for females. Dodds et al. comprehensively reviewed the literature on normative data for HGS and found that the data followed a similar pattern to the British normative data, with a rise during infancy, maintenance during midlife, and a decline from midlife. They also reported that the HGS was significantly lower in developing countries than developed nations [6]. Based on these findings, they recommend using region-specific cut-offs for HGS in various countries [26].

We analyzed the women in our study with an HGS below 16 kg, as recommended by EGWSOP2, to diagnose probable sarcopenia. Of these women, 62.26% were postmenopausal, while 37.73% were premenopausal. However, among the women who did not have sarcopenia, 48.14% were premenopausal and 51.85% were postmenopausal. These two percentages were comparable.

Our analysis revealed a high incidence of probable sarcopenia in Saudi women using a cut-off of 16. Approximately 40% of all women had a handgrip strength (HGS) of less than 16 kg. When the HGS 16 cut-off was applied to the premenopausal and postmenopausal groups separately, it was found that the prevalence of probable sarcopenia was 44% in postmenopausal women and almost 34% in premenopausal women. 

We used data from Saudi premenopausal women with non-obesity to establish handgrip strength cut-off values for identifying low grip strength in postmenopausal women. These cut-offs were determined using the suggested values of [mean −2 SDs] and [mean −2.5 SDs] from the EGWSOP2 consensus. An HGS of 10 kg is the cut-off point proposed to identify probable sarcopenia in Saudi women, and an HGS of 8 kg is a conservative measure for this purpose. These values are lower than the cut-off values found in other populations, such as Turkey [27], Italy [28], Spain [29], and Britain [6], which highlights the differences in population anthropometrics.

In a study similar to ours, Bhat et al. reported HGS cut-offs in Turkey using EWGSOP2’s method to determine low HGS as mean −2.5 SDs of young Turkish adults as a reference group and compared these Turkish-specific values to other published population-specific cut-offs. The Turkish study included 114 females, and the mean HGS of young Turkish women was 33.10 (SD ± 5.30) kg, and the HGS was 22.5 kg and 19.8 kg when the HGS cut-off was calculated as mean minus 2 SDs and mean minus 2.5 SDs of the young reference women, respectively [27]. Individuals from diverse geographic locations exhibited varying levels of HGS, with the highest values observed in Europe and North America, the lowest in Africa, South Asia, and South East Asia, and intermediate in China, South America, and the Middle East [30].

The handgrip strength test is vital in determining frailty and predicting all-cause mortality, cardiovascular mortality, and morbidity. It is quick, inexpensive, and simple to conduct, making it a promising tool for stratifying an individual’s risk of disease. However, published reference ranges for handgrip strength are mostly based on Caucasian individuals, with limited data on non-Caucasian populations [4,30]. 

Several methodologies have been employed in the literature to estimate handgrip strength cut-offs for identifying low strength. Studies conducted in Finland, Italy, and Turkey have established an HGS threshold, derived from receiver operating characteristic (ROC) analysis, that predicts mobility limitations or slow gait speed [28,31,32]. 

In the literature, it has been conventional to employ statistical methods, such as dichotomization, to categorize patients into low and normal appendicular skeletal muscle mass index (ASMI) based on various cut-off values. However, this method may obscure true dose–response relationships, and limitations in analyzing these variables must be addressed in future sarcopenia studies [33]. Consequently, muscle strength and muscle mass evaluated as continuous variables must be considered [33].

An Italian epidemiological study investigated the risk factors for mobility and disability related to aging in Tuscany [28]. The study analyzed how muscle function and calf area decline with age and impact mobility. They tested several sarcopenia indices, including handgrip, knee-extension isometric torque, lower extremity muscle power, and calf muscle area. For each index, sarcopenia was defined to be present when the value was <2 SDs below the mean. The prevalence of sarcopenia increased with age for all four indices, with a maximum gradient for muscle power and a minimum for calf muscle area. However, lower extremity muscle power was no better than handgrip or knee-extension torque in the early detection of poor mobility, defined as walking speed < 0.8 m/s or inability to walk at least 1 km without difficulty. They estimated strength cut-off values for clinical applications and recommended using HGS to diagnose sarcopenia [28]. Similarly, a Finnish study determined the optimal HGS cut-off value for predicting an increased risk of walking difficulties in individuals aged 55 and above. They found that the cut-off points for a higher probability of mobility limitation in men were 37 kg and 21 kg for women. Also, they evaluated whether these values varied based on BMI and discovered that handgrip strength cut-points increased with BMI in men, but not in women [32].

In this study, postmenopausal women had weaker handgrip strength than premenopausal women, suggesting a relationship between menopause and muscle strength. These observations support previous investigations indicating that the transition to menopause increases the risk of sarcopenia [34,35]. Muscle mass and strength may be associated with the decline in estrogen levels after menopause [36], as skeletal muscles have estrogen receptors, and estrogen may have an anabolic effect on these receptors [11]. Estrogen deficiency severely impairs the maintenance of satellite cells, or muscle stem cells, and their capacity to self-renew and differentiate into muscle fibers [37]. Preliminary results from a biopsy study of women transitioning from perimenopause to post-menopause indicate a loss of satellite cells concurrent with the decline in estradiol in women [37]. These results demonstrate the crucial function of estrogen in maintaining satellite cells and muscle regeneration in females [37]. Aging is also associated with a progressive deterioration in neuromuscular function caused by structural motor neuron changes. These changes include decreased number and size of neurons, reduced transmission at neuromuscular junctions, and decreased density of the junctional fold and active zone. These transformations decrease the number of motor units, their firing rate, contractile speed, specific tension, and stability across the neuromuscular junction [38].

Moreover, menopause at a young age may exacerbate the reduction in muscle strength since women are exposed to hypoestrogenism for an extended period. Four cross-sectional studies involving over 10,000 women have been systematically reviewed to examine the impact of physical function and menopause. The studies found that premature menopause before the age of 40 and early menopause before the age of 45 are associated with weaker grip strength, but the likelihood of functional decline decreases in menopause after age 50 [39]. Furthermore, the chair stand test was not affected by the age of menopause, underscoring the value of handgrip strength measurement in such evaluations [39]. Conversely, contradictory results were reported in a study of Canadian women, where early menopause was only associated with gait speed, not grip strength [40].

In Hispanic women, menopause before the age of 45 significantly increases the risk of developing sarcopenia, and the prevalence of those with low muscle strength evaluated by handgrip strength increases from 7.1% in women in their 40s to 79.4% in those over 80. Similarly, physical performance decreases from 0.5% in the younger group to 60.5% in the older group. The risk of sarcopenia rises sharply from 6.7% in Hispanic younger women to 58.1% in older women. Meanwhile, frailty affects less than 1% of Hispanic women under 60, rising to 39.5% in those over 80 [41].

An interesting investigation of Mexican-American women reported that differences between pre- and postmenopausal women in grip strength become insignificant after age adjustment [35]. However, it found that women who worked with their hands by grinding tortillas had better grip strength [35]. Postmenopausal women can benefit from physical activity to increase muscle strength [34], as it stimulates myofibrillar protein synthesis and inhibits protein breakdown [42]. Nevertheless, menopause-related hormonal changes can reduce daily physical activity, resulting in a sedentary lifestyle and lower energy expenditure [43,44]. A decline in handgrip strength among postmenopausal women can be attributed to estrogen deficiency and reduced physical activity. 

Due to the high prevalence of obesity among Saudi women, we compared the prevalence of probable sarcopenia between those with and without obesity. Using the EGSWOP2 cut-off of 16 kg, we discovered that probable sarcopenia was higher in the obesity group. Similarly, the new Saudi-specific cut-off of 10 kg showed a higher prevalence in the obesity group. However, when a more conservative cut-off of 8 kg was employed, the rates of probable sarcopenia were comparable between the obesity and non-obesity groups.

The significant differences between premenopausal and postmenopausal women in handgrip strength in our sample, with insignificant differences in muscle mass, could be attributed to several reasons, including bone mineral density, obesity, and menopause-related hormonal alterations. Evidence from the literature shows a positive correlation between handgrip strength and bone mineral density in premenopausal women [25], while no association is observed between grip strength and osteoporosis in postmenopausal women [45]. Nevertheless, they found a positive association between osteoporosis and muscle mass in postmenopausal women [45].

Although muscle mass strongly predicts handgrip strength [25], our analysis showed no significant differences in muscle mass indices between premenopausal and postmenopausal women. However, we found significant disparities in mass metrics, but not handgrip strength, when we divided the cohort into four groups based on obesity and menopausal status. Specifically, non-obesity groups exhibited lower appendicular skeletal muscle mass and appendicular skeletal muscle mass index values than the obesity groups. While obesity may mask the presence of sarcopenia due to a lower muscle mass percentage in relation to total body mass, similar to individuals without obesity, it is essential to note that individuals with obesity often have increased lean mass [46]. Therefore, employing body fat percentage from bioelectrical impedance analysis to define probable sarcopenia implies that body composition, rather than just body mass, is a relevant indicator of sarcopenia.

Obesity is an independent risk factor for decreased muscle strength [47], linked to metabolic derangements in adipose tissue that cause oxidative stress, inflammation, and insulin resistance [48]. These factors negatively impact muscle health [48], leading to the possibility that obesity could contribute to the independent decline of muscle mass and strength [49]. A sedentary lifestyle can contribute to both sarcopenia and obesity, exacerbating each other [49]. A meta-analysis suggests that obesity is the primary cause of functional deterioration rather than decreased muscle mass [50]. Obesity-related factors, such as inactivity, insulin resistance, and chronic inflammation, can lead to increased muscle fat deposition, known as myosteatosis [51]. Myosteatosis can reduce muscle strength, mobility, and overall survival and prognosis and can also influence sarcopenia outcomes, even without sarcopenia [52]. Myosteatosis is marked by an abundance of macrophages in muscle tissue that release pro-inflammatory cytokines, resulting in insulin resistance [53]. The accumulation of fat deposits in skeletal muscles is strongly associated with visceral adipose tissue, which is a predictor of insulin resistance [53]. Furthermore, the increased lipolysis of adipose tissue leads to an influx of free fatty acids into muscle cells, contributing to insulin resistance [53]. These mechanisms have been proposed to explain the connection between myosteatosis and sarcopenia consequences.

Losing ovarian hormones during menopause is associated with changes in adipose tissue distribution from subcutaneous to visceral regions [54].Consistent with our previous study [14],we found that postmenopausal women had higher BMI, percentage of total body fat, and fat mass than premenopausal women, which may be attributed to alterations in endogenous sex hormones as a potential mechanism of action to explain the increase in adiposity in women during menopause [55].

Sarcopenic obesity is a medical condition that occurs when excess body adiposity and sarcopenia coexist [49]. The cross-talk between adipose and muscle tissues suggests that obesity and sarcopenia can worsen each other [56]. Both premenopausal and postmenopausal women in our sample have high levels of adiposity, consistent with previous studies conducted on the Saudi population [57,58]. Our analysis showed that the averages of the overall body mass index (BMI) and body fat percentage (%BF) of the entire group were categorized as obesity according to both parameters [59].

Sex-specific differences in the development and progression of sarcopenia have been observed in older adults, with differences in the hormonal and cytokine/chemokine changes that occur [60]. In women, high IL-16 levels and obesity increase the risk of sarcopenia, whereas malnutrition and low IL-16 levels are risk factors in men. Studies have found that women with sarcopenia have higher IL-16 levels than controls, whereas men have lower levels, suggesting that chronic inflammation can cause sarcopenia [61]. Additionally, the severity of sarcopenia in women is linked to higher inflammation, as physical performance decline is associated with inflammatory markers [62].

After menopause, adipose tissue undergoes phenotypic changes due to metabolic inefficiencies in subcutaneous and visceral adipose tissue [63]. Abildgaard et al. examined these differences during menopause by collecting subcutaneous and visceral adipose tissue biopsies from pre-, peri-, and postmenopausal women aged 45–60. They found that postmenopausal women had larger adipocytes, more inflammation, and fibrosis in subcutaneous adipose tissue [63]. This was accompanied by an increase in visceral fat accumulation and a decrease in insulin sensitivity, which was associated with alterations in the morphology of the visceral adipose tissue [63].

The infiltration of fat tissue into muscle mass can result in micro changes in muscle composition and function, referred to as “muscle quality” [1,64]. These changes can affect muscle strength, are often used to indicate muscle quality [1], and are a crucial factor in determining clinical outcomes [65,66].

Premenopausal and postmenopausal women differ in age, making it challenging to separate the effects of aging from those of menopause. As noted in our previous study, age strongly predicts increased cardiovascular risk among women [14]. The mid-40s-to-50s transition to menopause is characterized by a considerable decrease in ovarian follicle counts and a corresponding decrease in estrogen levels [67]. While the effects of estrogen on muscle mass have been well documented [67], a recent study in Spain found no significant association between endogenous hormones and muscle strength in postmenopausal women aged 47 to 83 years [29]. The synergistic effect of aging and menopause on muscle tissue is still being studied. As muscle is more metabolically active than adipose tissues, ageing is associated with increased fat mass and decreased fat-free mass and basal metabolic rate [68].

Sarcopenic obesity is not limited to older adults and can affect younger adults and middle-aged people with obesity [49], particularly after weight loss interventions, and is associated with decreased muscle strength [69,70,71,72]. Historically, muscle strength has been measured in older individuals or patients; however, the paradigm shifts in sarcopenia to incorporate muscle strength rather than mass loss is still uncommon in young people. Therefore, we emphasize the importance of muscle strength testing in young, healthy adults to understand sarcopenia better.

When determining diagnostic cut-offs for any given measure, it is essential to consider how they relate to the measured parameter’s consequences. In our study, we explored the trend of handgrip strength in Saudi women, proposed cut-offs, and recommended using population-specific values when available. However, more research is needed to examine the proposed cut-offs concerning sarcopenia-related outcomes through long-term studies to confirm their usefulness as a marker of adverse consequences.

## 5. Strengths and Limitations

This study is the first to provide specific cut-offs for low handgrip strength in Saudi women to diagnose sarcopenia based on muscle strength. Our novel approach considers menopausal status. We focused on handgrip strength instead of muscle mass, as the latter is more closely related to functional decline and adverse outcomes. The primary outcomes were evaluated using widely used and validated measurements, including handgrip strength and bioelectrical impedance analysis, to assess muscle strength, appendicular skeletal muscle, and body composition. An additional advantage of the cut-off points is their practicality in clinical settings, as they are simple and easy to apply. We also developed new cut-offs using the EGWSOP2 algorithm, which fully meets the EGWSOP2 consensus on the definition and diagnosis of sarcopenia. This study’s cross-sectional design and small sample size are limitations that must be considered when interpreting the results, as they affect the study’s generalizability. Since the criteria for sarcopenia were derived from a small number of participants, further research with a larger cohort of Saudi non-sarcopenic young women without obesity is required to extend the findings to Saudi women. Our recommendation for future research is to assess the habitual physical activity level, which is crucial in determining muscle mass and strength. It would also provide valuable insight into Saudi women’s level of physical activity and whether they are as inactive as women in the United States and Europe.

## 6. Conclusions

The discrepancy in sarcopenia rates based on different cut-off values among Saudi postmenopausal women highlights the importance of using ethnic cut-points in clinical practice. We evaluated the impact of using the EWGSOP2 cut-off versus population-specific cut-offs on estimating the prevalence of probable sarcopenia, characterized by low handgrip strength, in Saudi women. Our study showed that the EWGSOP2 cut-off for low HGS diagnosis to detect sarcopenia in women is not adaptable to Saudi postmenopausal women. Although HGS was lower in Saudi postmenopausal women, Saudi premenopausal women were still diagnosed with low HGS using the current cut-offs. Developing new population-specific cut-offs from Saudi women may be clinically relevant and more adapted. However, before this new criterion is implemented in the clinical routine, future studies must fully comprehend these findings’ consequences and determine whether they correlate with declines in physical function.

## Figures and Tables

**Table 1:** Clinical parameters and sarcopenia indices by menopausal status.

Clinical Parameters	All (*n* = 134)	Premenopausal Women (*n* = 59)	Postmenopausal Women (*n* = 75)	
Anthropometrics	Mean ± SD	Mean ± SD	Mean ± SD	*p* Value
Age (years)	52.88 ± 11.65	42.63 ± 8.25	60.95 ± 6.40	˂0.001
Weight (kg)	77.11 ± 15.01	74.78 ± 15.82	78.95 ± 14.17	0.111
Height (cm)	156.47 ± 6.19	157.27 ± 6.12	155.84 ± 6.22	0.186
**Body Composition Indices**				
BMI (kg/m^2^)	31.52 ± 5.56	30.14 ± 5.45	32.61 ± 5.43	0.010
% FM	39.22 ± 5.56	37.64 ± 6.01	40.47 ± 4.87	0.003
FM (kg)	31.17 ± 9.65	29.30 ± 10.15	32.64 ± 9.03	0.046
FFM (kg)	45.84 ± 6.66	45.15 ± 7.48	46.39 ± 5.93	0.289
**Sarcopenia Indices**				
HGS (kg)	17.15 ± 4.79	18.12 ± 4.65	16.39 ± 4.79	0.037
ASM (kg)	18.66 ± 2.77	18.41 ± 3.02	18.87 ± 2.55	0.342
ASMI (kg/ht^2^)	7.63 ± 1.05	7.47 ± 1.07	7.76 ± 1.03	0.122

Note: Data are presented as mean ± SD; *p* < 0.05 is considered significant. Abbreviations: BMI: body mass index; %FM: fat mass percentage; FM: fat mass; FFM: fat-free mass; HGS: handgrip strength; ASM: appendicular skeletal muscle; ASMI: appendicular skeletal muscle index.

**Table 2:** Comparison of women’s clinical parameters and sarcopenia indices regarding menopausal and obesity status.

Clinical Parameters	Premenopausal Women with Non-Obesity (*n* = 30)	Premenopausal Women with Obesity (*n* = 29)	Postmenopausal Women with Non-Obesity (*n* = 28)	Postmenopausal Women with Obesity (*n* = 47)
	Mean ± SD	Mean ± SD	Mean ± SD	Mean ± SD
**Age (years)**	41.33 ± 9.88 *	43.97 ± 6.03	62.18 ± 7.59	60.21 ± 5.54 ^#^
**Weight (kg)**	63.17 ± 7.52 *	86.79 ± 12.89	65.29 ± 6.98	87.09 ± 10.65 ^#^
**Height (cm)**	155.97 ± 5.56	158.62 ± 6.46	156 ± 5.57	155.74 ± 6.64
**Body Composition Indices**				
**BMI (kg/m^2^)**	26.03 ± 2.71 *	34.38 ± 4.17	27.04 ± 2.06	35.94 ± 3.84 ^#^
**% FM**	33.47 ± 5.30 *	41.97 ± 2.78	36.46 ± 4.35	42.85 ± 3.39 ^#^
**FM (kg)**	21.70 ± 5.94 *	37.17 ± 7.13	24.11 ± 4.54	37.72 ± 6.96 ^#^
**FFM (kg)**	40.97 ± 4.62 *	49.48 ± 7.46	41.64 ± 3.53	49.21 ± 5.23 ^#^
**Sarcopenia Indices**				
**HGS (kg)**	18.83 ± 4.45	17.38 ± 4.81	16.04 ± 4.16	16.60 ± 5.17
**ASM (kg)**	16.50 ± 2.38 *	20.38 ± 2.27	16.75 ± 1.57	20.13 ± 2.16 ^#^
**ASMI (kg/ht^2^)**	6.80 ± 0.76 *	8.17 ± 0.88	6.86 ± 0.59	8.30 ± 0.85 ^#^

Note: Data are presented as mean ± SD; *p* < 0.05 is considered significant. Abbreviations: BMI: body mass index; %FM: fat mass percentage; FM: fat mass; FFM: fat-free mass; HGS: handgrip strength; ASM: appendicular skeletal muscle; ASMI: appendicular skeletal muscle index. * *p* < 0.001 versus Premenopausal Women with Obesity. ^#^ *p* < 0.001 versus Postmenopausal Women with Obesity.

**Table 3:** Prevalence of probable sarcopenia in Saudi women using EWGSOP2 definition.

Categories	Total (*n* = 134)	Premenopausal Women (*n* = 59)	Postmenopausal Women (*n* = 75)
**Probable Sarcopenia**	53 (39.55%)	20 (37.73%)	33 (62.26%)
**Non-Sarcopenia**	81 (60.45%)	39 (48.14%)	42 (51.85%)

Note: According to the EWGSOP2 definition, in women, probable sarcopenia is identified as handgrip strength < 16 kg, and non-sarcopenia is identified as handgrip strength ≥ 16 kg [1]. Data are presented as numbers (percentage; %). Abbreviations: EWGSOP2: European Working Group on Sarcopenia in Older People 2 [1]. Chi-square *p*-value is >0.05.

**Table 4:** Handgrip strength cut-off points to identify low handgrip strength (HGS) in postmenopausal women were calculated from [mean −2 SD] and [mean −2.5 SD] of the premenopausal Saudi women reference population.

HGS (kg)		
Mean	Mean −2 SD	Mean −2.5 SD
18.83 ^a^	10 ^b^	8 ^b^

Note: ^a^ represents the mean of HGS of the premenopausal Saudi women reference population. ^b^ Figures are rounded for practical application. Abbreviations: HGS: handgrip strength; SD: standard deviation.

## Data Availability

The data presented in this study are available on reasonable request from the corresponding author.
